# Rapid Grapevine Health Diagnosis Based on Digital Imaging and Deep Learning

**DOI:** 10.3390/plants13010135

**Published:** 2024-01-03

**Authors:** Osama Elsherbiny, Ahmed Elaraby, Mohammad Alahmadi, Mosab Hamdan, Jianmin Gao

**Affiliations:** 1 School of Agricultural Engineering, Jiangsu University, Zhenjiang 212013, China; osama_algazeery@mans.edu.eg; 2 Agricultural Engineering Department, Faculty of Agriculture, Mansoura University, Mansoura 35516, Egypt; 3 Department of Cybersecurity, College of Engineering and Information Technology, Buraydah Private Colleges, Buraydah 51418, Saudi Arabia; ahmed.elaraby@svu.edu.eg; 4 Department of Computer Science, Faculty of Computers and Information, South Valley University, Qena 83523, Egypt; 5 Department of Software Engineering, College of Computer Science and Engineering, University of Jeddah, Jeddah 23890, Saudi Arabia; mdalahmadi@uj.edu.sa; 6 Interdisciplinary Research Center for Intelligent Secure Systems, King Fahd University of Petroleum and Minerals, Dhahran 31261, Saudi Arabia; mosab.mohamed@kfupm.edu.sa

**Keywords:** grapevine disease, digital imaging, GLCM, hybrid deep networks, transfer learning, standalone software

## Abstract

Deep learning plays a vital role in precise grapevine disease detection, yet practical applications for farmer assistance are scarce despite promising results. The objective of this research is to develop an intelligent approach, supported by user-friendly, open-source software named AI GrapeCare (Version 1, created by Osama Elsherbiny). This approach utilizes RGB imagery and hybrid deep networks for the detection and prevention of grapevine diseases. Exploring the optimal deep learning architecture involved combining convolutional neural networks (CNNs), long short-term memory (LSTM), deep neural networks (DNNs), and transfer learning networks (including VGG16, VGG19, ResNet50, and ResNet101V2). A gray level co-occurrence matrix (GLCM) was employed to measure the textural characteristics. The plant disease detection platform (PDD) created a dataset of real-life grape leaf images from vineyards to improve plant disease identification. A data augmentation technique was applied to address the issue of limited images. Subsequently, the augmented dataset was used to train the models and enhance their capability to accurately identify and classify plant diseases in real-world scenarios. The analyzed outcomes indicated that the combined CNN_RGB_-LSTM_GLCM_ deep network, based on the VGG16 pretrained network and data augmentation, outperformed the separate deep network and nonaugmented version features. Its validation accuracy, classification precision, recall, and F-measure are all 96.6%, with a 93.4% intersection over union and a loss of 0.123. Furthermore, the software developed through the proposed approach holds great promise as a rapid tool for diagnosing grapevine diseases in less than one minute. The framework of the study shows potential for future expansion to include various types of trees. This capability can assist farmers in early detection of tree diseases, enabling them to implement preventive measures.

## 1. Introduction

Grapes are a globally important crop, with a significant economic impact [1]. The existence of diseases in grapevines poses a severe threat to global food security, as they significantly contribute to crop losses ranging from 10 to 30% [2]. Vines are highly susceptible to a wide variety of fungal diseases that can reduce yields. These include leaf spot (*Isariopsis griseola*), gray mold (*Botrytis cinerea*), downy mildew (*Plasmopara viticola*), powdery mildew (*Erysiphe necator*), esca (*Phaeomoniella chlamydospora* and *Phaeoacremonium aleophilum*), and black rot (*Guignardia bidwellii*). All of these diseases have an adverse effect on plant leaves or the crop itself, which can lead to a moderate to extreme loss in production of one or both [3]. The health of grapevines can be affected by a variety of factors, with stress being induced by both biotic and abiotic elements. Biotic stress arises from live pathogens such as fungi, viruses, and bacteria, which are the most prevalent pathogenic agents [4]. In contrast, abiotic stress is linked to non-living factors such as climate and soil conditions. For instance, chlorosis is often a physiological symptom, not a disease in itself. It is caused by factors such as nutrient deficiencies (iron, nitrogen, magnesium, or zinc), poor soil conditions (inadequate drainage, high pH, or compacted soil), or environmental stress (water imbalance, root damage, or exposure to pollutants), all of which lead to the yellowing of leaves [5,6]. The most prevalent method of mitigating biotic stress in grapevines involves the administration of chemical substances. While this method has proven to be extremely successful, it can also have a detrimental impact on the environment and overall agricultural revenue, as it is not always a cost-effective strategy [7]. Numerous precision agriculture techniques have been developed in response to the aforementioned factors in an effort to maximize agricultural output while decreasing the impact of external factors such as pests and disease [7]. As a result, the agricultural industry makes extensive use of distant and proximal sensing techniques, as well as big data technologies, computer vision, robots, deep learning (DL) and machine learning (ML) techniques, and high-performance computers. These methods have utility beyond plant disease diagnosis, encompassing weed detection, crop quality assessment, yield prediction, species identification, water and soil monitoring, and irrigation system management [7,8].

The success of classification algorithms and digital imaging techniques depends on a number of factors, for example, transfer learning, characteristics of the training inputs, data augmentation, and the combination of multiple trained deep networks. To solve classification issues with a small dataset, ML practitioners might turn to transfer learning, a technique that employs previously trained networks—typically those with deep architectures [9]. Using this strategy, the original pretraining weights are preserved. They are partially updated as new data are brought to the network. The main point of this method is to take advantage of the deep learning network’s prior learning from training models to make it easier to train a new, related classification issue that does not use the same feature space or distribution [10]. Multiple studies confirmed that using deep learning, particularly transfer learning, effectively classifies plant diseases, achieving over 80% accuracy [11,12,13]. This approach reduces computational time and enables the training of diverse classes with a substantial number of instances, making it particularly suitable for deep architectures. A variety of well-established pretrained networks, such as AlexNet, GoogleNet, ResNet, and the VGG family, are available. These DL models exhibit variations in their layer architectures. In transfer learning, typically only the final layer’s parameters are adjusted, while the rest of the architecture extracts features from training samples.

Texture features based on the gray level co-occurrence matrix (GLCM) are also crucial for identifying diseases in grapevines. Jaisakthi et al. [14] developed a system for detecting grapevine diseases, focusing on extracting texture features such as color variations after segmentation, to classify diseases effectively using support vector machine (SVM), adaboost, and random forest (RF) algorithms. This method, distinguished by its high accuracy in diagnosing diseases like rot and leaf blight, highlights its value in improving agricultural disease management. Data augmentation, considered during the training phase in this work, is an additional factor that can influence the performance of the detection model. Augmenting data by applying a sequence of transformations—such as mirroring or rotating an image—increases the dataset’s usefulness and depth [15]. Moreover, deep network fusion was extensively employed to enhance the quality and durability of the sickness detection model. Previously, this approach had been utilized for identifying plant phenotypes. Xiao et al. [16] employed a convolutional neural network (CNN) with the Resnet50 architecture to successfully detect various strawberry diseases, including gray mold, crown leaf blight, fruit leaf blight, powdery mildew, and leaf blight, utilizing datasets comprising both original and feature-enhanced images. Similarly, Koklu et al. [17] achieved impressive classification performance by generating a CNN-SVM model, extracting features from the Logits layer of the MobileNetv2 architecture, and employing various SVM kernels to classify the leaves of grapevines into five different species.

Traditionally, DL models in agriculture have relied on single-modal data, particularly plant images. However, advanced agricultural practices have led to a growing trend of exploiting multimodal data, which combine plant images with additional features like GLCM variables and pretrained characteristics. This shift has the potential to improve accuracy and performance in estimating plant phenotypes by incorporating diverse data sources. The current study distinguishes itself from other research in the field by exploring innovative deep networks, along with the creation of standalone software designed for a first-level model. Hence, the primary objectives of this research were (i) to construct a well-organized hybrid deep network to detect illnesses in grapevines using high-level characteristics extracted from RGB and GLCM, (ii) to develop an independent, easy-to-use software solution named AI GrapeCare for the quick assessment and analysis of digital imagery related to grapevine disease spread, (iii) to explain the superior components of a deep network for robust detection of grapevine infections, (iv) to examine the behavior of deep networks in different scenarios involving both augmented and non-augmented data, and (v) to compare the performance of various hybrid deep networks that combine CNNs and deep neural networks (DNNs) with long short-term memory (LSTM), as well as applying pre-trained features such as VGG16, VGG19, ResNet50, and ResNet101V2 during training. All of these procedures aim to select the best model that can be recommended for precision agriculture in the future.

## 2. Materials and Methods

### 2.1. Image Database

**Figure 1:**
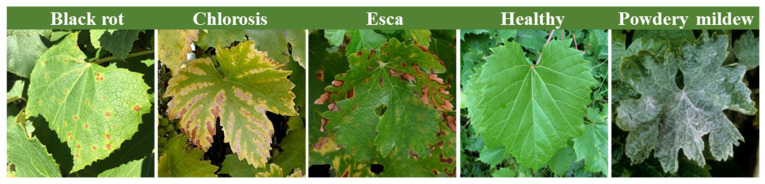
Illustrative samples of the five primary grapevine diseases.

To overcome the limitations of the PlantVillage database for real-life applications, the disease detection platform (PDD) datasets were created to support the plant. These datasets are accessible at pdd.jinr.ru, accessed on 10 December 2023 [18]. The PDD database differs from PlantVillage as it encompasses diverse conditions, angles, and backgrounds. This study utilized a dataset of PDD images to discover diseases in grapevines, specifically including real-life images of grape leaves affected by diseases. These images were captured directly from grape-growing regions. As illustrated in Figure 1, the database comprises a total of 295 images, which are separated into five distinct categories of grape crops. These classes consist of 31 images depicting black rot, 49 with chlorosis, 73 affected by esca, 121 healthy samples, and 22 showing powdery mildew. All images included in the database contain relevant information about both the crops and the diseases present on them, with a standardized size of 256 × 256 pixels. Additionally, it compiles images of various symptoms of each disease, enabling us to differentiate diseases even when their symptoms appear similar.

### 2.2. Image Preprocessing Techniques

To ensure effective model training and to minimize any defects that may arise during the imaging process, it is necessary to perform RGB image preprocessing before proceeding with data analysis. The preprocessing phase entails various stages, including segmentation to eliminate any background elements, data augmentation to increase the size of the training dataset, and feature transformation to normalize and standardize the characteristics of the images. First, performing background separation is essential to isolate the grapevine plant and eliminate any extraneous elements from the images. A segmentation process called the threshold technique, which involves converting the image to grayscale and producing a binary image, was utilized [19]. The image has two possible pixel values: a value equal to 1, representing grapevine pixels, and a value equal to 0, signifying non-grapevine pixels that may be excluded. The image is binary, with each pixel represented by a single bit. Once the grapevine pixels were separated from the background objects, a set of potential color features was obtained for further analysis. Second, data augmentation is a crucial step in enhancing the learning process of a deep network and for enabling it to identify objects in images captured under diverse real-world conditions. In this work, several augmentation techniques were performed to increase confidence in the categorization process. These included using the original image, applying a zoom range of 0.3, rotating the image up to 90 degrees, flipping it horizontally, and shifting its width by a range of 0.1 and its height by a range of 0.2. Third, to address variations in feature magnitudes, normalization is applied to each feature individually. The normalization calculation is derived by dividing the range between the maximum and minimum feature values by the minimum image data value.

### 2.3. Texture Characteristics Derived from the Gray Level Co-Occurrence Matrix

**Figure 2:**
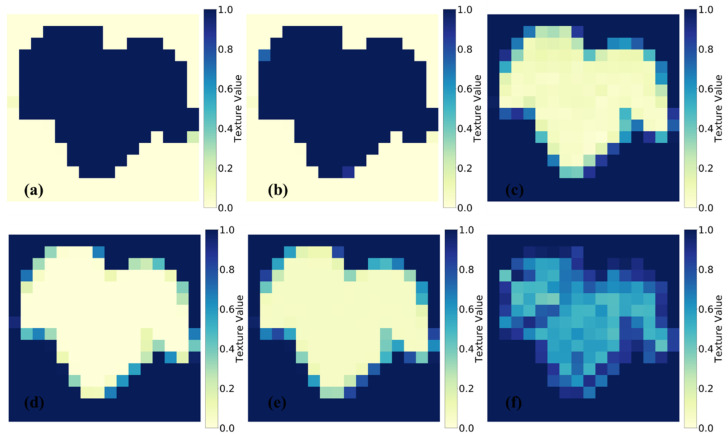
GLCM-based texture characteristic analysis of segmented healthy grape leaves: (**a**) contrast, (**b**) dissimilarity, (**c**) homogeneity, (**d**) ASM, (**e**) energy, and (**f**) correlation.

The use of the gray level co-occurrence matrix (GLCM) for detecting and diagnosing plant diseases has been extensively studied in various research works. Yogeshwari and Thailambal [20] employed a GLCM as a feature extraction method in their framework for detecting plant leaf diseases, successfully capturing texture details from the images. Sari et al. [21] employed texture characteristics and an SVM model for the classification of chili leaf disease. The co-occurrence matrix is a statistical technique employed for the analysis of texture in a grayscale image [22]. By examining the grayscale correlation between two pixels in the image space separated by a specific distance, the GLCM extracts the texture features of the canopy through probability characteristics in a random manner. This study involved six different versions of the GLCM, namely, contrast, dissimilarity, homogeneity, angular second moment (ASM), energy, and correlation, as feature extraction techniques. The depth and texture of an image are indicated by its contrast, whereas dissimilarity calculates the separation between pairs of pixels within a specified area. Homogeneity evaluates how close the distribution of elements in the GLCM is, while ASM detects the roughness of the distribution and texture of the image. Energy is a measurement of the uniformity of texture, and correlation determines the degree of correlation present in the local grayscale image. As depicted in Figure 2, this work utilizes the GLCM methodology to extract texture information from segmented grape leaves. The GLCM algorithm computes texture characteristics for each pixel region that plays a crucial role in subsequent analyses. The following formulas, as described in Hall-Beyer [23], provide a detailed explanation of the specific variables derived from the RGB-based GLCM approach:

(1) $$Contrast=\sum_{i,j=0}^{N-1}P_{i,j}{\left(i-j\right)}^{2}$$

(2) $$Dissimilarity=\sum_{i,j=0}^{N-1}P_{i,j}|i-j|$$

(3) $$Homogeneity=\sum_{i,j=0}^{N-1}\frac{P_{i,j}}{{1+(i-j)}^{2}}$$

(4) $$ASM=\sum_{i,j=0}^{N-1}{P_{i,j}}^{2}$$

(5) $$Energy=\sqrt{ASM}$$

(6) $$Correlation=\sum_{i,j=0}^{N-1}P_{i,j}\left[\frac{\left(i-\mu_{i})((j-\mu_{j}\right)}{\sigma_{i}\sigma_{j}}\right]$$

where $P_{i,j}$ represents the probabilities calculated for values where *i* and *j* serve as row and column indices, respectively. The mean values for indices i and *j* are denoted by $\mu_{i}$ and $\mu_{j}$, respectively. The standard deviation for values in reference to i and j indices are given by $\sigma_{i}$ and $\sigma_{j}$, respectively. *N* signifies the total number of rows or columns.

### 2.4. Overview of the Suggested Approach

**Figure 3:**
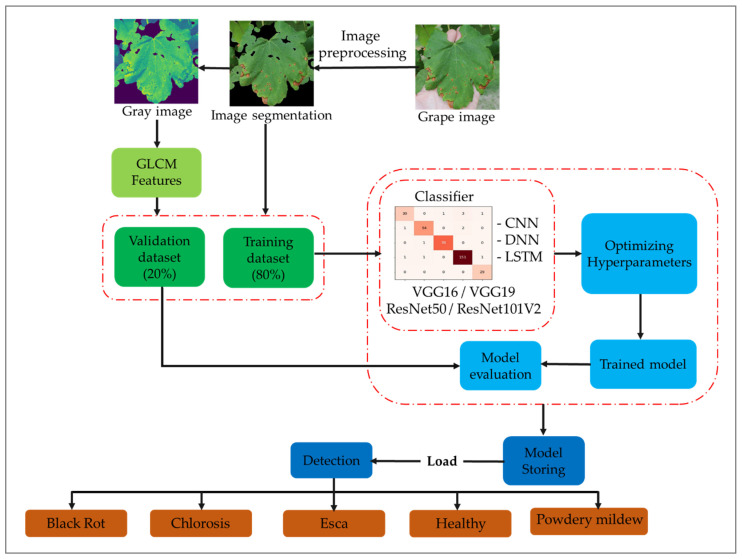
Schematic of the methodology utilized in the present study.

Multiple deep networks, comprising CNN, DNN, CNN-LSTM, and DNN-LSTM architectures, underwent training using a diverse range of multimodal data, which included GLCM variables, pre-trained features, and image data. Pre-trained characteristics were obtained from VGG16, VGG19, ResNet50, and ResNet101V2. Generally, CNN and DNN were trained using image data, while LSTM utilized GLCM factors for disease identification in grapevine crops, for example black rot, chlorosis, esca, powdery mildew, and healthy. The proposed framework’s sequential steps are depicted in Figure 3: (a) perform image preprocessing tasks such as normalization, segmentation, and augmentation; (b) convert the images to grayscale to extract GLCM features; (c) divide the dataset and train deep neural networks; (d) train the models under different scenarios, including CNN, DNN, CNN-LSTM, and DNN-LSTM; (e) incorporate pre-trained features extracted from VGG16, VGG19, ResNet50, and ResNet101V2 during the training process; (f) optimize hyperparameters to select the best configuration during training; (g) analyze the model’s performance on new samples; (h) evaluate the classification model’s overall performance; (i) save the advanced model for future use; and (j) load the advanced network model to support grape disease managers in making effective decisions.

### 2.5. Deep Networks

#### 2.5.1. Deep Neural Network (DNN)

**Figure 4:**
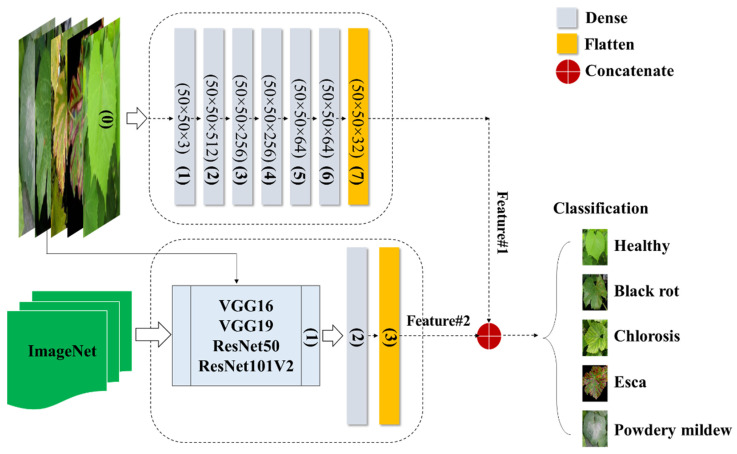
Fusion of DNNimg structure and pretrained networks.

According to Schmidhuber [24], a DNN is a type of neural network characterized by multiple hidden layers and thousands of neuron units, each performing simple computations. The DNN model is recognized for its powerful and fundamental deep learning architecture [25]. The model’s neural network layers can be divided into three components: the input layer, hidden layers, and output layer. The input layer of the classification method incorporates all the features present in the constructed feature space. To predict the classified image, six implicit layers are incorporated. Through multiple trials, the model was structured with six hidden layers, where the numbers of neurons in each hidden layer were set as 512, 256, 256, 64, 64, and 32 in a sequential manner. The model was trained with a batch size of 5, employing the Adabelta optimizer, and underwent a total of 100 epochs. Figure 4 illustrates the creation of the DNN model, which is built upon image data and incorporates transfer-based learning characteristics. Furthermore, all the extracted features were combined to form a new attribute for observing a specific class within a set of 5 categories.

#### 2.5.2. Convolutional Neural Network (CNN)

**Figure 5:**
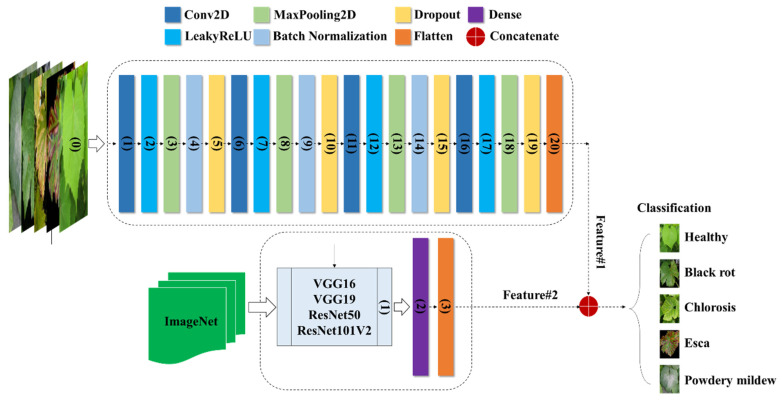
Integration of the CNNimg architecture with transfer learning networks.

CNN is a popular deep learning method used for tasks such as object recognition, image classification, and regression. Its effectiveness is evident through numerous successful applications utilizing 2D feedforward architectures [26]. The CNN architecture is composed of three main types of layers: (1) the input layer, which contains the primary data for the deep network, like image data; (2) hidden layers, which include convolutional, max-pooling, and flattened layers; and (3) the output layer, a fully connected layer that flattens and sends inputs from other layers. Our framework also includes additional layers such as dense layers, dropout layers, and leaky rectified linear units (LeakyReLU). To adjust their input with a set of filters, convolutional layers (CLs) modify their input during the model optimization process. The dense layer organizes neurons and accepts information from all previous layers. Dense layers are employed to identify images based on CL output. Dropout layers help prevent overfitting by setting the outgoing edges of hidden units (neurons that make up hidden layers) to zero during each update of the training phase. LeakyReLU layers use a threshold operation that involves multiplying any input value less than zero by a fixed scalar. The max-pooling layer picks the maximum element from the filter’s feature map region, producing an output feature map that contains the most prominent features of the previous feature map. Applying a max-pooling layer of size (2, 2) to the recovered matrix helped solve the overfitting problem. The last fully connected layers act as classifiers via the softmax function and are implemented using the dense class. As illustrated in Figure 5, the CNN configuration comprises two distinct models employing various transfer learning approaches to train image data for the detection of grapevine diseases. The first model directly incorporates image data (Table 1), while the components of the second model (Table 2) rely on pretrained feature transfer.

#### 2.5.3. Long Short-Term Memory (LSTM)

LSTM is a useful technique for identifying long-term dependencies and overcoming issues with vanishing and exploding gradients. This is achieved through the use of three gates: a forget gate that removes information, an input gate that stores cell states, and an output gate that sends cell states to the next cell. To implement LSTM, several steps must be taken, including data preprocessing, data splitting, and LSTM modelling [27]. The training process involves training multiple network models on the training data and selecting the best model based on the smallest error value among the various training models. This is achieved by calculating the value of each gate function for a maximum number of epochs or until the target error is reached. The training process is carried out within a cell that contains the three gates. These gates enable the network to selectively remember or forget information from previous time steps, allowing it to handle long-term dependencies more effectively than other types of recurrent neural networks. During training, the model is optimized to minimize the error between its predictions and the actual values in the training data. Achieving the desired outcome involves the adjustment of the network’s weights and biases through backpropagation and gradient descent, alongside the utilization of a designated loss function for error evaluation. The training process continues for a specified number of epochs or until a target level of performance is completed.

**Figure 6:**
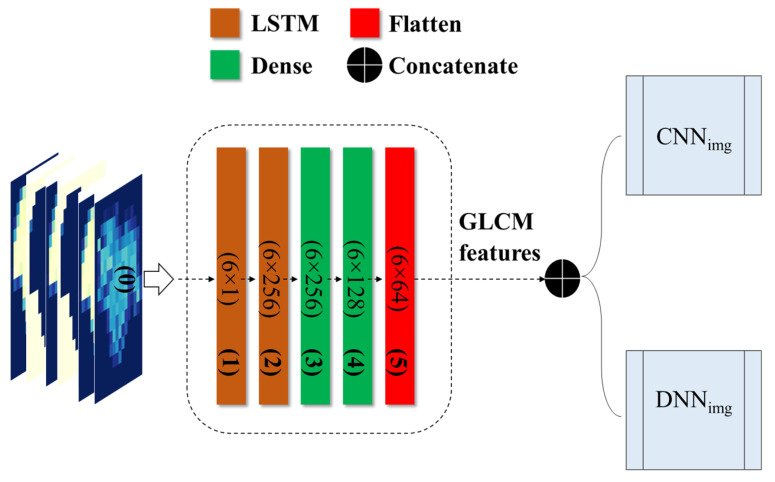
Flowchart of the LSTM architecture integrating GLCM-based characteristics with the proposed deep networks.

The grapevine health status detection model, based on LSTM, was constructed utilizing GLCM variables. The components of the model are elaborated in Figure 6. The LSTM model-based features can be integrated with different deep networks, such as CNN_img_ and DNN_img_, creating a novel hybrid network. In an LSTM model, the dense layer connects all neurons from the previous layer and changes the dimension of a vector. It uses matrix-vector multiplication and can be trained and updated through backpropagation. The dense layer is typically used for the output layers and employs the softmax activation function to assign probabilities to different classes. The final prediction is based on the category with the highest probability.

### 2.6. Dataset and Model Training

To avoid memory constraints, each plant image in the study was scaled down to 50 × 50 pixels. A total of 295 nonaugmented and 1770 augmented images were divided into 80% (236 and 1416 samples) for training and 20% (59 and 354 samples) for validation. The Kaggle platform, which provides free access to NVidia K80 GPUs in kernels, was utilized to achieve a 12.5X speedup in deep learning model training. The platform was tested on a PC with an Intel Core i7-3630QM processor running at 2.4 GHz and 8 GB of RAM. The TensorFlow library version 2.6.2 was used to implement DNN, CNN, and LSTM modules for the classification task.

### 2.7. AI GrapeCare Software

**Figure 7:**
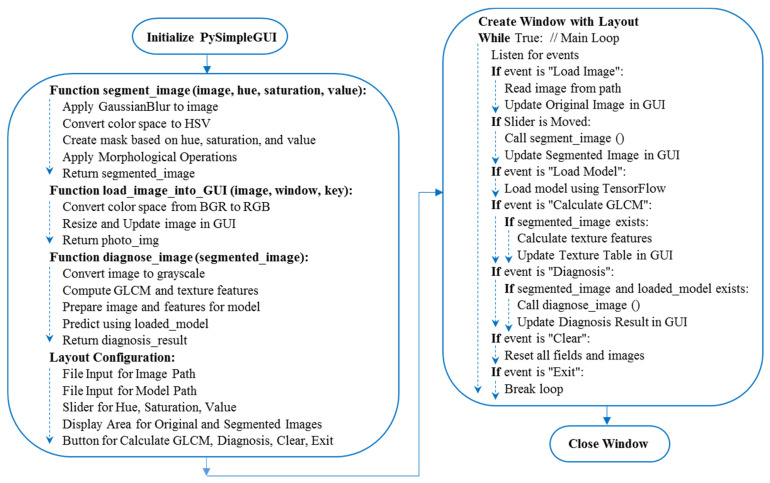
Pseudo-code for constructing the graphical user interface (GUI) of the AI GrapeCare software and diagnosing the health state of grapes.

The GrapeCare AI software, specialized in digital image analysis of grape health, was built on Python version 3.8.8. It harnesses a range of Python libraries for various functionalities. For instance, OpenCV [28] and SciKit-Image [29] are the backbone for image processing tasks. Numerical operations are handled by NumPy [30], while TensorFlow Keras [31] is incorporated for ML applications, particularly for deploying pre-trained diagnostic models. The software’s graphical interface is designed using PySimpleGUI, offering a user-friendly way to create and present the user interface [32]. To ensure cross-platform compatibility, including Windows, Linux, and Mac OS, PyInstaller [33] was applied. The Python script, the hybrid deep network that was generated, grape disease samples from real-world conditions, and the stand-alone version of this software are all available for download on Google Drive (https://drive.google.com/file/d/1uOVAMiFDWBZsm8U9alzSdSk2c-A8zWBN, accessed on 10 December 2023), packaged in a RAR file with a size of 1.03 GB. As depicted in the overarching flowchart (Figure 7), the pseudo-code explains the establishment of the AI GrapeCare software and its associated functions. The software workflow is organized into five primary stages: (1) loading the RGB image, (2) uploading the trained deep network model, (3) preprocessing the images (e.g., background segmentation), (4) extracting phenotypic features, and (5) assessing grape health conditions.

### 2.8. Performance Evaluation

To assess the classification performance, a quantitative analysis was employed in this study. Evaluation metrics, including precision (*Pr*), recall (*Re*), overall accuracy (*Acc*), intersection over union (*IoU*), and F-measure (*Fm*), were utilized to compare and examine the classification performance. These metrics were calculated for each image in the validation dataset, and average values were computed across all images, where *FN* stands for false negatives, *FP* for false positives, *TN* for true negatives, and *TP* for true positives.

(7) $$Pr=\frac{\sum TP}{\sum TP+\sum FP}\times 100$$

(8) $$Re=\frac{\sum TP}{\sum TP+\sum FN}\times 100$$

(9) $$Acc=\frac{\sum TP+\sum TN}{\sum TP+\sum TN+\sum FP+\sum FN}\times 100$$

(10) $$IoU=\frac{TP}{FP+TP+FN}\times 100$$

(11) $$Fm=2\times \left(\frac{Pr\times Re}{Pr+Re}\right)\times 100$$

## 3. Results and Discussion

### 3.1. Pretrained Network-Based DNN Model

In this study, we evaluated the performance of DNNs in recognizing various types of grapevine diseases using both augmented and non-augmented versions, along with a variety of transfer learning networks, including VGG16, VGG19, ResNet50, and ResNet101V2. The results, encompassing performance measures such as Pr, Re, Fm, and IoU, as well as validation and training outcomes (Acc and Ls), are presented in Table 3. The DNN model outputs achieved high accuracy with the VGG16 network and augmented data. The findings confirmed that the DNN_RGB_-VGG16-1770 model demonstrated superior performance in identifying grapevine disease, with a Pr of 0.929, Re of 0.927, Fm of 0.925, and IoU of 0.863. The validation values for Ls and Acc were determined to be 0.258 and 0.927, respectively. The DNN-based model demonstrated a robust correlation between the selected features and the overall health of the plants, further solidifying its efficacy in disease detection and monitoring. Alternatively, the network of DNN_RGB_-ResNet101V2-295 exhibited lower performance (Pr = 0.382, Re = 0.610, Fm = 0.467, and IoU = 0.439) in assessing the health of grapevines. The validation values for Acc and Ls were 0.610 and 1.291, respectively. This model was less effective in accurately identifying and evaluating the overall health status of the grapevine compared to the DNN_RGB_-VGG16 model. The analysis results provided further insight into the influence of three key factors on the behavior of deep networks. First, the process of filtering high-level features during training was observed to impact the network’s performance [34]. Second, the specific neural network architecture chosen for the task played a significant role in shaping the network’s behavior [35]. Third, augmented data significantly contributes to the improvement of the proposed models [8].

### 3.2. Pretrained Network-Based CNN Model

To enhance detection performance and ensure accurate identification of grapevine diseases, transfer learning features (VGG16, VGG19, ResNet50, and ResNet101V2) were seamlessly integrated with a characteristics-based CNN. Additionally, the dataset was expanded through the implementation of data augmentation techniques. Table 4 presents the results of evaluating CNN models for diagnosing grapevine disease, comparing various performance measures (Pr, Re, Fm, and IoU) across the models. Also, the behavior of the models was controlled by employing training and validation procedures, which involved closely monitoring model loss and accuracy. Among the transfer learning networks and models evaluated, the augmented version of CNN_RGB_-VGG16-1770 demonstrated superior performance compared to the nonaugmented CNN_RGB_-VGG16-295 model and other transfer learning networks. The measured classification capabilities for Pr, Re, Fm, and IoU were recorded as 0.955, 0.955, 0.955, and 0.914, respectively. Furthermore, the validation outcomes indicated an accuracy of 0.955 and a model loss of 0.151. The results are consistent with the findings of Nagi and Tripathy [36], who confirmed the reliability of CNN in diagnosing grapevine diseases, highlighting its remarkable capabilities in accurately identifying and classifying various diseases. This underscores the significant contribution of CNN in supporting disease diagnosis within the field of viticulture.

### 3.3. Fusion of DNN-LSTM Model with Pretrained Networks

Various approaches of integrating deep networks were explored and investigated to identify the most effective model for evaluating grapevine health. These approaches involved integrating deep networks, specifically by fusing pretrained networks (VGG16, VGG19, ResNet50, and ResNet101V2) with DNN-LSTM. The established model via DNN-LSTM, in combination with VGG16 and augmented data, demonstrated exceptional performance compared to other networks. As presented in Table 5, the model’s classification yielded the following metrics: Pr = 0.924, Re = 0.924, Fm = 0.923, and IoU = 0.858. Additionally, the validation results displayed an accuracy of 0.924 and a loss of 0.265. Alternatively, the hybrid network of DNN_RGB_-LSTM-ResNet101V2-295 demonstrated subpar performance (Pr = 0.365, Re = 0.593, Fm = 0.451, and IoU = 0.422) in evaluating the health of grapevines. The corresponding validation values for Acc and Ls were 0.593 and 1.268, respectively. Moreover, we inspected hybrid models that combined DNN_RGB_-LSTM_GLCM_ with VGG16-295, VGG19-1770, ResNet101V2-295, and ResNet101V2-1770 and observed their superior performance compared to a single DNN network. The findings emphasize the key role of precise model construction and strategic network selection in achieving superior performance in deep networks [37].

### 3.4. Top-Level Deep Network: CNN-LSTM with Pretrained Networks

The distinctive characteristics of the CNN_img_-LSTM_GLCM_ model, coupled with the utilization of transfer learning networks, had a crucial role in effectively and accurately identifying and classifying various types of grapevine diseases. The integrated model consists of two composite models: an image data-based CNN model, alongside a GLCM-based LSTM model. The inclusion of these models has significantly improved the performance of the deep network fusion, as it encompassed the key variables necessary for the accurate classification of grapevine health. Table 6 provides insights into the evaluation and comparison of the performance of the model constructed (CNN_img_-LSTM_GLCM_) using proposed transfer learning networks. The CNN_RGB_-LSTM_GLCM_-VGG16 model achieved exceptional scores across all metrics, with Pr, Re, and Fm at 0.966 and IoU at 0.934. Improved validation results with an Acc of 0.966 and reduced Ls of 0.123 confirm the network’s prominent role in grapevine health assessment. The VGG16-based hybrid network, empowered by data augmentation, proved to be an effective model for grapevine health assessment. Its exceptional architecture and rich features propelled it to outperform all other approaches [8], setting a new benchmark in vineyard diagnostics.

### 3.5. Learning Curves for Hybrid Deep Network Analysis

**Figure 8:**
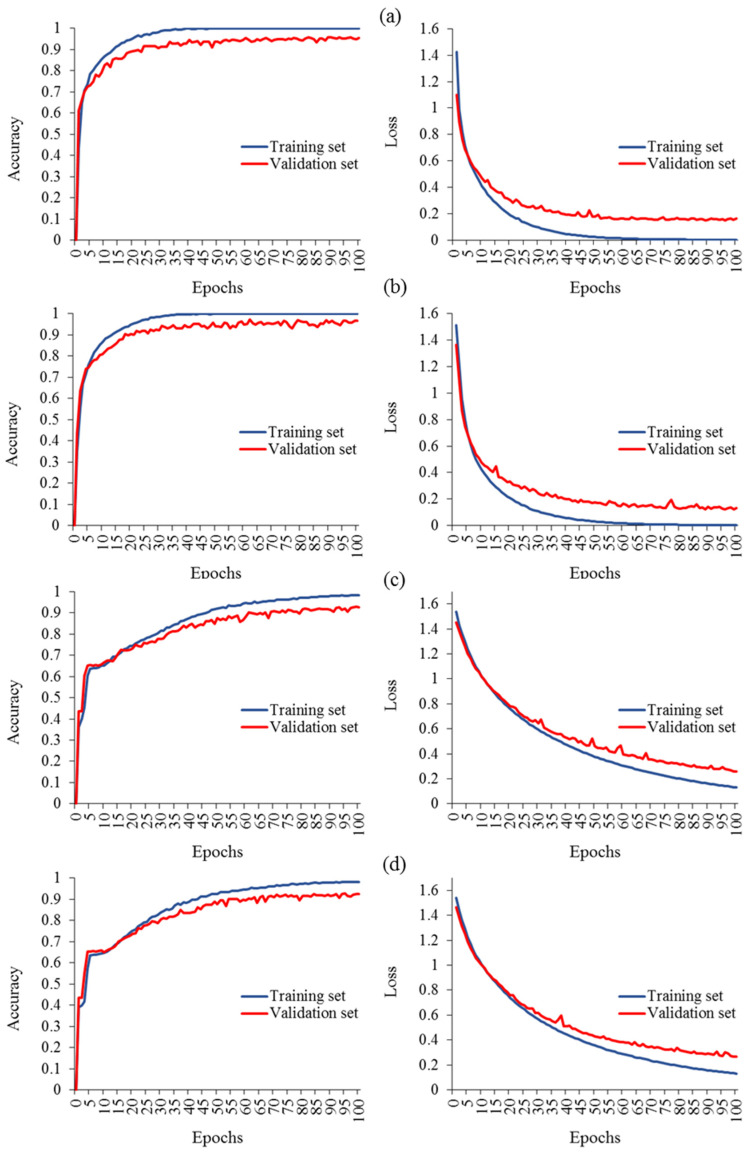
Evaluation measures for diverse hybrid deep networks: (**a**) CNN_img_-VGG16, (**b**) CNN_img_-LSTM_GLCM_-VGG16, (**c**) DNN_img_-VGG16, and (**d**) DNN_img_-LSTM_GLCM_-VGG16.

Several investigations have effectively improved the learning curve of classification models through various approaches, for instance, augmenting the dataset, optimizing parameter selection, refining the deep network architecture components, leveraging pretrained features, integrating multimodal data including GLCM features, and combining different DL models (e.g., DNN-LSTM and CNN-LSTM). After implementing these strategies, novel hybrid deep networks were successfully developed. Figure 8 presents a comparative analysis of the created models, aiming to identify the model with the best learning curve. In terms of performance, the advanced models with augmented data versions exhibited remarkable learning curves, resulting in validation accuracies of 96.6% (Ls = 0.123), 95.5% (Ls = 0.151), 92.7% (Ls = 0.258), and 92.4% (Ls = 0.265) for the CNN_img_-LSTM_GLCM_-VGG16 (Figure 8b), CNN_img_-VGG16 (Figure 8a), DNN_img_-VGG16 (Figure 8c), and DNN_img_-LSTM_GLCM_-VGG16 (Figure 8d) models, respectively. The learning curves of the models exhibit favorable behavior, with both the training and validation accuracy gradually increasing over epochs. Simultaneously, the loss steadily decreases for both the training and validation data. The RGB-based CNN model, combined with the transfer learning network of VGG16, and the LSTM model based on GLCM variables demonstrated remarkable effectiveness in evaluating the health status of grapevines compared to other pretrained networks. The CNN_img_-LSTM_GLCM_-VGG16 model, developed in the third stage by integrating the three models, achieved outstanding classification performance with a Pr, Re, and Fm of 96.6% and an IoU of 93.4%. The learning curve for this model demonstrated outstanding results, consistent with the findings of Elmetwalli et al. [38]. Their work focused on enriching the expected performance by implementing various strategies to mitigate overfitting in the neural network. These strategies included incorporating an early stopping mechanism to prevent overtraining, filtering out high-level features, and optimizing the hyperparameters of the model. These measures were specifically designed to enhance the overall performance and improve the generalization capability of the neural network.

### 3.6. Analyzing Deep Network Performance via the Confusion Matrix

**Figure 9:**
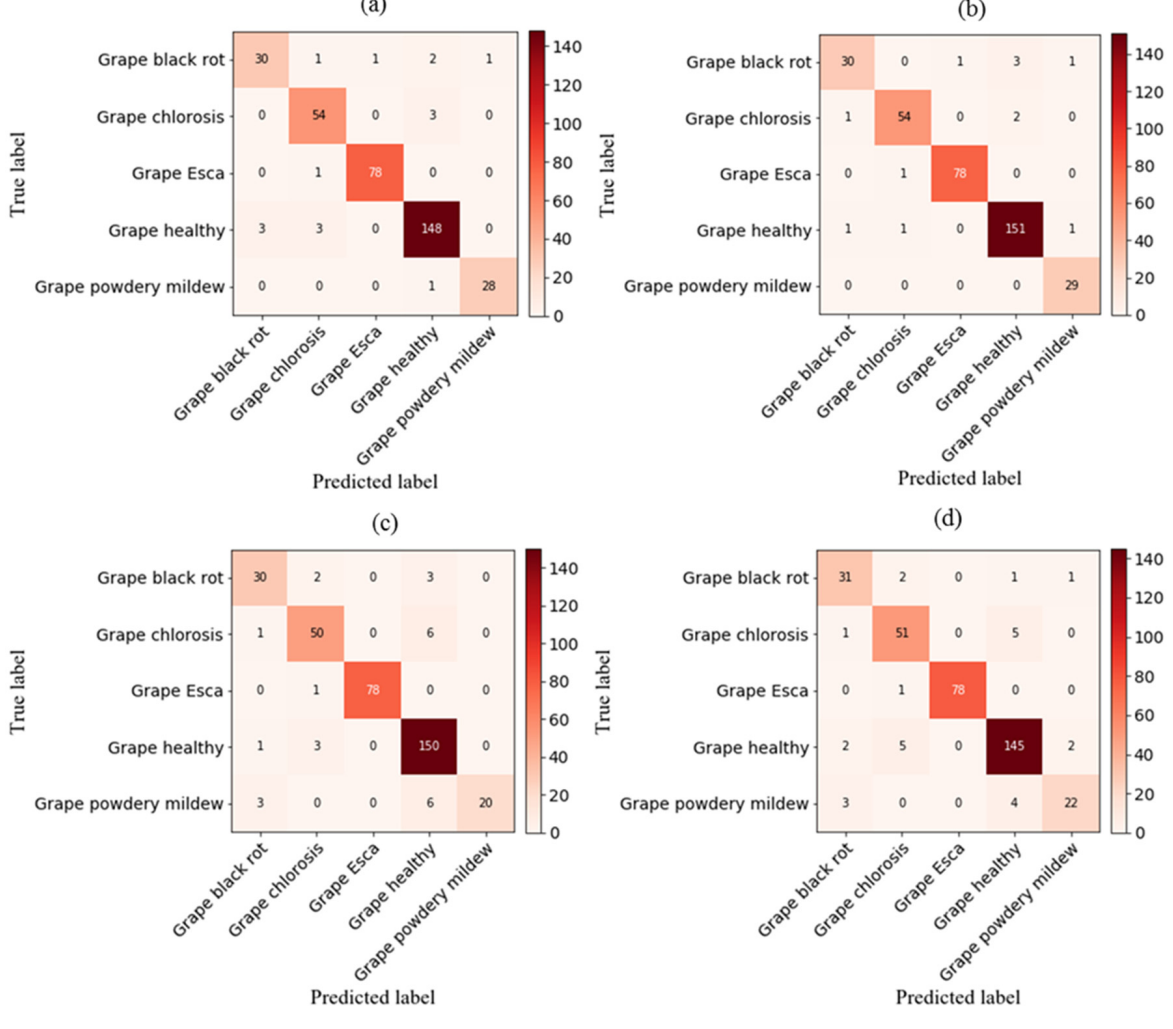
Confusion matrix for grapevine disease detection with various hybrid deep networks: (**a**) CNN_img_-VGG16, (**b**) CNN_img_-LSTM_GLCM_-VGG16, (**c**) DNN_img_-VGG16, and (**d**) DNN_img_-LSTM_GLCM_-VGG16.

As illustrated in Figure 9, the validation phase of the proposed CNN_RGB_-VGG16, CNN_RGB_-LSTM_GLCM_-VGG16, DNN_RGB_-VGG16, and DNN_RGB_-LSTM_GLCM_-VGG16 models for grapevine disease classification is depicted through a confusion matrix. In a validation dataset comprising 354 images, an analysis revealed that the first, second, third, and fourth models misclassified approximately 16, 12, 26, and 27 images, respectively. The developed CNN_RGB_-LSTM_GLCM_-VGG16 network outperformed the other proposed networks. It consistently exhibited superior true-positive and true-negative values, with a minimal number of false-negative and false-positive values. Hence, this proposed system effectively classifies various grapevine diseases. Due to its exceptional performance compared to previous studies, it represents a suitable method for modelling the health status of grapevines. Our findings demonstrated superior accuracy in comparison to the study conducted by Goncharov et al. [39], wherein they devised a profound Siamese convolutional network to mitigate the constraints arising from the scarcity of image databases. The results exhibited remarkable potential, as an accuracy of 92% was achieved in the precise identification of esca, black rot, and chlorosis diseases affecting grape plants. In addition, the model developed in this research exhibited exceptional performance, surpassing the findings of Ghoury et al. [40], who investigated disease detection in grapes using faster R-CNN. With a dataset made up of 260 images representing two disease classifications, they achieved a classification accuracy of 95.57%. Moreover, in comparison to Hasan et al. [41], the first-order model exhibited notable accuracy. Their research focused on the application of CNN for the recognition and classification of grape leaf diseases. CNN consists of three stages: data input, feature learning, and classification. In their study, they implemented CNN using Python and Keras libraries, resulting in an impressive accuracy rate of 91.37% and a learning rate of 0.0001.

### 3.7. AI GrapeCare: Software for Grape Health Analysis

**Figure 10:**
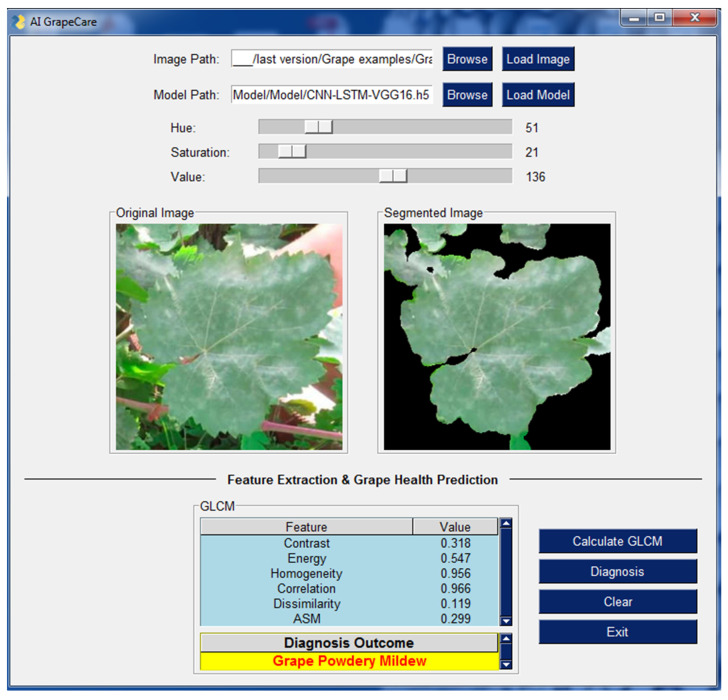
Outcomes of AI GrapeCare software during the test of one sample of grapevine.

As shown in Figure 10, the software interface is user-friendly and does not require any programming skills. The software is capable of loading and analyzing digital images with various extensions, such as PNG, JPG, JPEG, and TIFF. Additionally, it supports uploading a superior hybrid deep model named CNN_img_-LSTM_GLCM_-VGG16. The software includes image pre-processing procedures, for example image segmentation and compression. During image segmentation, AI GrapeCare software can aid users in selecting the optimal parameters by displaying visualizations of the segmented images it generates. Useful characteristics (textural features) can then be extracted and displayed in a table-based GUI, ready to be used as inputs for the model to assess the state of grape growth. The diagnosis time of the AI GrapeCare software for assessing grape health conditions is typically just a few seconds and never exceeds 1 min. The software’s model performance was recorded at 96.6%, with a loss of 0.123.

This study introduces a novel hybrid approach driven by a GUI for the real-time monitoring of grapevine health using RGB-based remote sensing techniques. Its practicality and cost-effectiveness make it a valuable solution for disease detection in plants. Innovative modelling techniques are employed to accurately identify the health status of plants through image analysis. The developed advanced model demonstrates exceptional performance in classifying diseases and reducing losses, indicating its effectiveness. Integrating UAV-based systems, which utilize high spatial resolution remote sensing, can enhance the monitoring of large-scale agricultural areas and significantly improve disease management. Additionally, the implementation of IoT-based RGB data transmission systems enables a nondestructive and efficient approach for monitoring grapevine health and diagnosing diseases. This technology facilitates timely data collection, minimizes plant disruption, and expedites the processing of remote sensing data, leading to faster and more informed decision-making in precision agriculture. The valuable insights from this study contribute to ongoing efforts to implement effective agricultural practices. By leveraging advancements in digital imaging and deep learning, this research contributes to the development of automated solutions that enhance disease detection and improve agricultural management efficiency. 

Finally, although this research can detect chlorosis in grapevines, it is important to note that chlorosis can result from various factors, as mentioned in the literature. Therefore, future studies should focus on developing a model capable of effectively recognizing chlorosis and understanding its underlying causes as a symptom. This is crucial due to the multifaceted nature of the manifestation, which poses significant challenges in accurate identification. By concentrating on this aspect, researchers can better address the complexities involved in specifying chlorosis. In addition, our developed methodology is proficient in precisely classifying individual diseases on leaves. However, there is an imperative need to advance diagnostic techniques that can distinguish and simultaneously discover various pathogens exhibiting alike symptoms on the same leaf. Implementing object detection algorithms with two or more classes can be instrumental in these examination improvements, providing a layered and comprehensive understanding of vine pathology.

## 4. Conclusions

Grapevine, a globally significant fruit crop, is often plagued by four prevalent diseases: esca, chlorosis, powdery mildew, and black rot. Timely and accurate diagnosis plays a pivotal role in containing the spread of diseases and minimizing production losses in the grapevine. Progress in deep learning has paved the way for innovative diagnostic algorithms in the field of plant disease identification, unlocking new possibilities and avenues for accurate and efficient detection. This paper introduces an innovative hybrid approach, utilizing stand-alone software named AI Grapecare, to process and analyze RGB images for identifying grapevine diseases. The proposed framework integrates various deep learning networks, including a convolutional neural network (CNN), long short-term memory (LSTM), and a deep neural network (DNN). It also employs multimodal features like VGG16, VGG19, ResNet50, and ResNet101V2, along with texture characteristics based on the gray level co-occurrence matrix (GLCM). The hybrid models, such as CNN_img_-VGG16, CNN_img_-LSTM_GLCM_-VGG16, DNN_img_-VGG16, and DNN_img_-LSTM_GLCM_-VGG16, were trained with 80% of the data, while the remaining 20% was reserved for evaluation. This methodology allowed for a thorough performance assessment, enabling the selection of the most effective model. Based on experimental outcomes, the hybrid network of CNN_img_-LSTM_GLCM_-VGG16 exhibited remarkable predictive capability as a feature extractor and classifier for grapevine disease diagnosis. By incorporating its extracted features, the proposed system achieved impressive classification performance, with precision, recall, and F-measure reaching 96.6% and an intersection over union of 93.4%. The system’s validation accuracy was 96.6% with a loss of 0.123. The proposed AI GrapeCare-based approach demonstrated the superiority of the chosen model architecture, highlighting its effectiveness in classifying grapevine disease leaves with minimal processing time and labor involvement. AI GrapeCare is expected to enhance phenotyping efficiency in precision agriculture by analyzing fruit health dynamics, leading to the consistent cultivation of high-quality crops.

## Figures and Tables

**Table 1:** Components of the proposed deep CNN structure.

Layer	Type	Input Size	Layer	Type	Input Size
0	Input data	(50 × 50 × 3)	11	Conv2D	(6 × 6 × 512)
1	Conv2D	(50 × 50 × 3)	12	LeakyReLU	(6 × 6 × 64)
2	LeakyReLU	(50 × 50 × 1024)	13	MaxPooling2D	(6 × 6 × 64)
3	MaxPooling2D	(50 × 50 × 1024)	14	Batch Normalization	(2 × 2 × 64)
4	Batch Normalization	(17 × 17 × 1024)	15	Dropout	(2 × 2 × 64)
5	Dropout	(17 × 17 × 1024)	16	Conv2D	(2 × 2 × 64)
6	Conv2D	(17 × 17 × 1024)	17	LeakyReLU	(2 × 2 × 64)
7	LeakyReLU	(17 × 17 × 512)	18	MaxPooling2D	(2 × 2 × 64)
8	MaxPooling2D	(17 × 17 × 512)	19	Dropout	(1 × 1 × 64)
9	Batch Normalization	(6 × 6 × 512)	20	Flatten	(1 × 1 × 64)
10	Dropout	(6 × 6 × 512)	-	-	-

**Table 2:** Elements of the proposed structure for transfer learning networks.

Layer	Input Data	Transfer Learning	Dense	Flatten
Input size	(50 × 50 × 3)	(50 × 50 × 3)	(1 × 1 × 512)	(1 × 1 × 256)

**Table 3:** The expected outcomes of DNN models, with and without data augmentation, using various reinitialized deep networks.

Model	Features	Augmented	Training			Validation		Performance			
			Acc	Ls	Tt	Acc	Ls	Pr	Re	Fm	IoU
DNN_img_	VGG16	Yes	0.982	0.126	13.172	0.927	0.258	0.929	0.927	0.925	0.863
		No	0.792	0.712	4.192	0.695	0.925	0.559	0.695	0.601	0.532
	VGG19	Yes	0.981	0.125	14.412	0.915	0.297	0.916	0.915	0.915	0.844
		No	0.835	0.645	6.526	0.678	0.853	0.543	0.678	0.579	0.513
	ResNet50	Yes	0.876	0.406	24.446	0.788	0.558	0.788	0.788	0.777	0.650
		No	0.682	0.990	7.642	0.627	1.191	0.546	0.627	0.517	0.457
	ResNet101V2	Yes	0.754	0.629	37.695	0.709	0.729	0.695	0.709	0.688	0.549
		No	0.631	1.121	8.650	0.610	1.291	0.382	0.610	0.467	0.439

**Table 4:** The expected outcomes of CNN models, with and without data augmentation, using various reinitialized deep networks.

Model	Features	Augmented	Training			Validation		Performance			
			Acc	Ls	Tt	Acc	Ls	Pr	Re	Fm	IoU
CNN_img_	VGG16	Yes	1.0	0.001	23.419	0.955	0.151	0.955	0.955	0.955	0.914
		No	0.987	0.095	5.403	0.746	0.588	0.744	0.746	0.731	0.595
	VGG19	Yes	1.0	0.005	23.481	0.932	0.224	0.932	0.932	0.931	0.873
		No	0.987	0.128	6.468	0.780	0.547	0.783	0.780	0.766	0.639
	ResNet50	Yes	0.851	0.454	30.968	0.701	0.787	0.689	0.701	0.689	0.539
		No	0.665	0.965	6.610	0.576	1.336	0.476	0.576	0.517	0.405
	ResNet101V2	Yes	0.645	0.961	47.838	0.559	1.154	0.547	0.559	0.544	0.388
		No	0.419	1.435	7.986	0.424	1.449	0.478	0.424	0.405	0.269

**Table 5:** The expected outcomes of DNN-LSTM models, with and without data augmentation, using various reinitialized deep networks.

Model	Features	Augmented	Training			Validation		Performance			
			Acc	Ls	Tt	Acc	Ls	Pr	Re	Fm	IoU
DNN_img_-LSTM_GLCM_	VGG16	Yes	0.982	0.127	14.443	0.924	0.265	0.924	0.924	0.923	0.858
		No	0.801	0.684	4.860	0.712	0.884	0.571	0.712	0.620	0.553
	VGG19	Yes	0.984	0.129	16.522	0.924	0.266	0.923	0.924	0.923	0.858
		No	0.818	0.669	7.207	0.712	0.883	0.723	0.712	0.638	0.553
	ResNet50	Yes	0.884	0.409	27.493	0.785	0.578	0.782	0.785	0.774	0.647
		No	0.665	1.009	7.255	0.593	1.201	0.363	0.593	0.450	0.422
	ResNet101V2	Yes	0.777	0.609	39.401	0.720	0.725	0.705	0.720	0.701	0.563
		No	0.627	1.126	9.695	0.593	1.268	0.365	0.593	0.451	0.422

**Table 6:** The expected outcomes of CNN-LSTM models, with and without data augmentation, using various reinitialized deep networks.

Model	Features	Augmented	Training			Validation		Performance			
			Acc	Ls	Tt	Acc	Ls	Pr	Re	Fm	IoU
CNN_img_-LSTM_GLCM_	VGG16	Yes	1.0	0.002	23.446	0.966	0.123	0.966	0.966	0.966	0.934
		No	0.992	0.102	5.509	0.712	0.658	0.678	0.712	0.678	0.553
	VGG19	Yes	1.0	0.004	28.189	0.929	0.226	0.930	0.929	0.929	0.868
		No	0.983	0.127	6.223	0.763	0.597	0.759	0.763	0.750	0.616
	ResNet50	Yes	0.852	0.469	34.549	0.732	0.727	0.726	0.732	0.718	0.577
		No	0.686	0.908	7.728	0.458	1.367	0.405	0.458	0.418	0.297
	ResNet101V2	Yes	0.655	0.946	45.575	0.579	1.060	0.567	0.579	0.552	0.408
		No	0.419	1.475	8.715	0.339	1.545	0.287	0.339	0.300	0.204

## Data Availability

The study data are available within the article.
